# Perioperative clinical and economic outcomes associated with replacing first-generation high molecular weight hydroxyethyl starch (Hextend®) with low molecular weight hydroxyethyl starch (Voluven®) at a large medical center

**DOI:** 10.1186/s13741-015-0013-0

**Published:** 2015-02-26

**Authors:** Raquel R Bartz, William D White, Tong J Gan

**Affiliations:** Department of Anesthesiology, Duke University Medical Center, Durham, NC USA; Department of Anesthesiology, Stony Brook University, HSC Level 4, Rm 060, Stony Brook, NY 11794-8480 USA

**Keywords:** Hetastarch, Perioperative fluid administration, Hextend®, Voluven®

## Abstract

**Background:**

Several plasma volume expander alternatives exist to enhance intravascular volume status in patients undergoing surgery. The optimal intravascular volume expander in the perioperative setting is currently unknown. Low molecular weight hetastarch, Voluven® (130/0.4), may have a better safety profile than high molecular weight hetastarch, Hextend® (450/0.7). We examined the clinical and cost outcomes of converting from Hextend® to Voluven® in a large tertiary medical center.

**Methods:**

Using a large electronic database, we retrospectively compared two different time periods (2009 and 2010) where the availability of semisynthetic colloids changed. Perioperative and postoperative outcomes including the use of red blood cells (RBC), platelets and coagulation factors, length of stay in the postoperative acute care unit (PACU), intensive care unit and hospital, as well as 30-day and 1-year mortality were compared. In addition, direct acquisition costs of all intraoperative and PACU colloids and crystalloid use were determined.

**Results:**

A total of 4,888 adult subjects were compared of which 1,878 received Hextend® (pre-conversion) and 2,759 received Voluven® (post-conversion) during two separate 7-month periods within 1 year apart, with the remainder receiving Plasmanate. The patients were similar in terms of patient demographics, preoperative comorbidities, ASA status, emergency surgery, types of surgery, intraoperative, and PACU times. In unadjusted outcomes, patients in the Hextend® group received more lactated Ringer’s than in the Voluven® group (2,220 + 1,312 vs. 1,946 ± 1,097 ml; *P* < 0.0001). The use of albumin (Plasmanate) was reduced from 10.5% of patients to 1.1% when Voluven® was substituted for Hextend®. Unadjusted outcomes were similar in each group including hospital LOS, percent change from baseline creatinine and receipt of intraoperative and PACU blood product administration. However, overall unadjusted total fluid costs were greater in the Voluven® compared to Hextend® group ($116.7 compared to $59.3; *P* < 0.001).

**Conclusions:**

Conversion from Hextend® to Voluven® in the perioperative period resulted in decreased albumin use and was not associated with changes in clinical outcomes and short- and long-term mortality. The conversion was associated with decreases in crystalloid use and an increase in colloid use and hence IV fluid acquisition costs in the Voluven® group.

## Background

Both human and semisynthetic colloids have been successfully used to increase intravascular volume in the perioperative period [1-4]. However, recent trials of volume replacement in the intensive care unit (ICU) have questioned the safety and efficacy of both human and synthetic colloids with one trial suggesting no benefit of albumin over saline for resuscitation and others suggesting harm with the use of semisynthetic colloids in this patient population [5-8]. The issue of safety has not been directly resolved in the perioperative setting. And, in many institutions, semisynthetic colloids are still available for use in patients undergoing surgical procedures where a benefit in decreased costs associated with intraoperative volume replacement may exist.

One of these semisynthetic products, Voluven®, was recently introduced into the US market. It has a lower molecular weight (150 kD) and molar substitution (0.4) and therefore has a theoretical lower plasma accumulation and tissue storage [9,10]. Previous investigations of Voluven® suggest less impact on factor VIII and Von Willebrand factor concentrations with a sustained plasma volume expansion effect resulting in less disturbance of coagulation with subsequent reduced blood loss and blood transfusion when compared to older larger molecular weight (MW) hydroxyethyl starch (HES) solutions [11,12]. In addition, up to 50 ml/kg of Voluven® can be given (about 3,500 ml in a 70-kg adult) compared to a maximum of 1,500 ml (20 ml/kg) of high MW hydroxyethyl starch to adult patients [13,14]. Therefore, Voluven® has the potential to reduce the need for other more costly plasma volume expanders such as 5% albumin as well as the need for blood transfusion. However, because of recent recommendations against the use of these products in ICU patients by regulatory agencies, hospitals have to evaluate the rationale for maintaining their availability for use in the perioperative period.

Additionally, anesthesiologists in the US are limited to using isotonic crystalloids and human albumin-based products (albumin or Plasmanate) in patients requiring intravascular volume expansion. Because of these institutional shifts of colloid types, hospital formulary costs will change. Currently, there is paucity of data that have examined the impact to hospital costs related to the use of different intravascular volume expanders. Therefore, we investigated two different periods evaluating the clinical and direct acquisition cost impact of converting from Hextend® to Voluven® in a large tertiary medical center. By comparing cases in which some colloid was given during two different periods, we sought to determine what differences in overall perioperative volume use, overall fluid costs, and certain perioperative outcomes might be associated with the use of the two different colloids.

## Methods

We conducted a retrospective comparison of two different time periods at a large academic medical center, Duke University Medical Center (DUMC). In March 2010, DUMC converted from Hextend® (hetastarch 6%, MW 670 kD, in balanced electrolyte solution) to Voluven® (hydroxyethyl tetrastarch 6% in sodium chloride 0.9%) as the semisynthetic colloid for use in the perioperative period. After Duke University Medical Center Institutional Review Board approval, de-identified data was obtained from an electronic anesthesia database which consists of physiologic and treatment characteristics used for perioperative care of the patient. Because de-identified clinical data was used, this study was determined to be exempt from patient consent. Two separate 7-month periods were investigated from 1 June 2009 to 30 December 2009 (Hextend®) and 1 June 2010 to 30 December 2010 (Voluven®). Anesthesia and surgical practice was otherwise similar in these two time periods; however, overall surgical volume increased due to increased operating room capacity in 2010. We avoided the 3-month interval before and after the conversion to ensure a period of stability in practice. Pre- and postoperative data were obtained by procedure ICD-9 codes, and our local institutional data warehouse known as the Decision Support Repositories was then linked to perioperative anesthesia records. Follow-up closed for both periods on 31 December 2012. All sequential cases greater than 18 years old, who received Hextend®, Voluven®, or albumin (Plasmanate®), were examined for inclusion. Cases in which <500 ml of Hextend® or Voluven® were recorded were not included in the analysis.

Patient demographics including gender, age, surgical comorbidities, type of surgery, duration of surgery, and type of anesthesia were obtained. Patient outcomes were compared between HES periods including volume of all crystalloids and colloids administered in the intraoperative and post-anesthesia care unit (PACU), the use of red blood cells (RBC) and platelets and coagulation factors. The length of stay in the PACU, hospital length of stay, intensive care unit length of stay, and 30-day and 1-year mortality were also compared. Fluid costs were calculated using the Red book acquisition costs of each product multiplied by the number of whole or partial units received intraoperatively or in the PACU. For 2013, these costs were Hextend® (500 ml) $37.80, Voluven® (500 ml) $61.06, Plasmanate (250 ml) $41.00, lactated Ringer’s (1,000 ml) $2.04, and 0.9% normal saline (1,000 ml) $2.65.

### Statistical analysis

Descriptive statistics including mean and standard deviation are presented for all measures, and mean values did not differ meaningfully form median. For the primary comparison of total fluid cost between periods, we used initial Wilcoxon rank-sum tests not subject to influence by extreme outliers, followed by multivariable regression analysis adjusting for BMI, duration of surgery, emergency status, regional or general anesthesia, and type of procedure. Unadjusted two-group tests were used to compare periods on other variables. For categorical demographic and outcome variables including gender, preoperative characteristics, type of anesthesia used, and death within 30 days and 1 year, the Pearson chi-square tests were used to determine *P* values, or the Pearson exact tests if counts were sparse. For comparisons of continuous measures and outcomes such as fluid volumes, length of stay, red blood cells used, and percent change creatinine, Wilcoxon rank-sum tests were used to determine *P* values. A *P* value <0.05 was considered statistically significant. All analyses were conducted on non-missing data; where data was incomplete, it was assumed to be consistent with non-missing data. SAS software version 9.3 (Cary, NC, USA) was used for statistical analysis.

## Results

Our database query for our defined sample initially returned a set of 5,548 cases. We then excluded 95 whose fluid volumes were zero and 196 aged <18 years old. We further excluded 206 who received <500 ml of Voluven® alone and 163 receiving <500 ml of Hextend® alone. The final overall analysis sample thus included 4,888 cases including 175 who received Plasmanate/albumin alone.

From 1 June 2009 to 30 December 2009, 2,098 were in the group in which Hextend® was the primary synthetic colloid and from 1 June 2010 to 30 December 2010, 2,790 patients were in the group in which Voluven® was available. An increase in surgical capacity accounts for the increased number of patients in the 2010 period. Otherwise, the patient demographics were similar in terms of patient characteristics, preoperative comorbidities, and emergency surgery with inconsequential differences in proportions of ASA status and types of surgery (Tables 1 and 2). Fewer patients in the 2010 group (90.1% vs. 93%, *P* < 0.001) received general anesthesia (Table 2). Intraoperative time and PACU time were similar between periods; however, in unadjusted outcomes, mean volume of lactated Ringer’s given differed significantly between periods; 2,252.9 ml in 2009 - Hextend® group vs. 1,958.9 in 2010 - Voluven® group; *P* < 0.0001. As expected, long-term follow-up also differed significantly between periods (Table 2). In 2009, 1,906 subjects received a mean of 767 ml (SD ± 301.8 ml) of Hextend® in the intraoperative period and 51 cases received a mean of 578.4 ml (SD ± 215 ml) in the PACU. The mean amount of Hextend® given for the entire operative and PACU period was 773.1 ml (SD ± 306.9 ml) compared to 933.7 ml (SD ± 518.4 ml) of Voluven® in 2010. Whereas, 2,650 subjects in the 2010 cohort received a mean of 905.8 ml (SD ± 495.5 ml) of Voluven® in the operative period and 263 subjects received 645.3 ml (SD ± 260.7 ml) of Voluven® in the PACU. Considering the volume of hetastarch, the mean volume of Hextend® received per case in 2009 was significantly lower than the mean volume of Voluven® received per case in 2010 (*P* < 0.001). The number of patients who received albumin, either alone or in combination with a perioperative product, decreased from 220 to 31 patients from the 2009 - Hextend® to 2010 - Voluven® period, although the mean volume of Plasmanate/albumin received per case did not change significantly between periods.

**Table 1:** **Patient characteristics**

	**2009 (** ***n*** **= 2,098)**			**2010 (** ***n*** **= 2,790)**			***P*** **value**
	**Total** ***N***	**Mean or** ***n***	**SD or percentage**	**Total** ***N***	**Mean or** ***n***	**SD or percentage**	
Age (yrs) (mean, SD)	2,098	58.1	15.4	2,790	58	15.6	0.8910
Female gender (*n*,%)	1,972	902	45.7	2,767	1,343	48.5	0.0574
Race (*n*,%)	1,966			2,762			0.7380
White		1,454	74		2,043	74	0.9933
Black		430	21.9		601	21.8	0.9266
Nat. Amer		23	1.2		26	0.9	0.4444
Asian		21	1.1		22	0.8	0.3322
Other		35	1.8		65	2.4	0.1621
Multiracial		3	0.2		5	0.2	1.0000
BMI (mean, SD)	1,884	29.6	7.3	2,639	29.5	7.3	0.9510
CAD preop (*n*,%)	1,383	721	52.1	1,839	1,016	55.2	0.0792
COPD preop (*n*,%)	1,383	83	6	1,839	116	6.3	0.7207
CABG preop (*n*,%)	1,383	9	0.7	1,839	12	0.7	0.9951
MI preop (*n*,%)	1,383	21	1.5	1,839	29	1.6	0.8942
Preop valve dz (*n*,%)	1,383	48	3.5	1,839	67	3.6	0.7938
Diabetes type I (*n*,%)	1,383	6	0.4	1,839	11	0.6	0.6274
Diabetes type II (*n*,%)	1,383	224	16.2	1,839	331	18	0.1799
Hypertension preop (*n*,%)	1,383	721	52.1	1,839	1,016	55.2	0.0792
Preop creatinine (mean, SD)	1,370	1	0.6	1,847	1.1	1.3	0.1725

yrs, years; Nat. Amer, Native American; BMI, body mass index; h/o, history of; CAD, coronary artery disease; COPD, chronic obstructive pulmonary disease; Preop valve dz, preoperative valve disease; CABG, coronary artery bypass graft; MI, myocardial infarction; DM, diabetes mellitus; preop, preoperative; SD, standard deviation.

**Table 2:** **Procedural characteristics**

	**2009 (** ***n*** **= 2,098)**			**2010 (** ***n*** **= 2,790)**			***P*** **value**
	**Total** ***N***	**Mean or** ***n***	**SD or percentage**	**Total** ***N***	**Mean or** ***n***	**SD or percentage**	
Emergency surgery (*n*,%)	1,878	149	7.9	2,677	226	8.4	0.5390
ASA class (*n*,%)	1,880			2,682			0.0045
1		48	2.6		59	2.2	0.4376
2		636	33.8		785	29.3	0.0011
3		1,074	57.1		1,612	60.1	0.0443
4		120	6.4		222	8.3	0.0168
5		2	0.1		4	0.1	1.0000
Procedure category	1,977			2,774			<0.0001
Misc. diagnostic (*n*,%)		4	0.2		10	0.4	0.4202
Obstetrical (*n*,%)		21	1.1		37	1.3	0.4007
Cardiovascular (*n*,%)		153	7.7		301	10.9	0.0003
Digestive (*n*,%)		398	20.1		530	19.1	0.3795
Ear (*n*,%)		32	1.6		73	2.6	0.0192
Endocrine (*n*,%)		17	0.9		33	1.2	0.2723
Eye (*n*,%)		3	0.2		4	0.1	1.0000
Female genital (*n*,%)		104	5.3		127	4.6	0.2811
Heme/lymphatic (*n*,%)		35	1.8		47	1.7	0.8427
Integumentary (*n*,%)		62	3.1		102	3.7	0.3141
Male genital (*n*,%)		104	5.3		104	3.7	0.0121
Musculoskeletal (*n*,%)		689	34.9		857	30.9	0.0041
Nervous system (*n*,%)		30	1.5		114	4.1	<0.0001
Nose, mouth, pharynx (*n*,%)		0	0		9	0.3	0.0129
Thoracic (*n*,%)		123	6.2		137	4.9	0.0553
Urinary (*n*,%)		139	7		199	7.2	0.8502
Other (*n*,%)		63	3.2		90	3.2	0.9115
General anes used (*n*,%)	1,977	1,848	93.5	2,774	2,499	90.1	<0.0001
Regional anes used (*n*,%)	1,977	647	32.7	2,774	930	33.5	0.5642
Surg. time (mins) (mean, SD)	1,505	240.8	128.6	2,399	242	131.8	0.8516
Rec’d any RBC, FFP, cryo (*n*,%)	1,977	440	22.3	2,774	578	20.8	0.2398
Days of follow-up (mean, SD)	1,976	1,081.6	323.6	2,774	776.7	211.6	<0.0001

Anes, anesthesia; SD, standard deviation; Misc, miscellaneous; Rec’d, received; RBC, red blood cell; Surg., surgical; Mins, minutes; FFP, fresh frozen plasma; Cryo, cryoprecipitate.

In unadjusted outcomes, we did not find a difference in blood product use in patients between periods; 22% of patients in 2009 and 21% in 2010 received a blood product which consisted of either a packed RBC, fresh frozen plasma, or cryoprecipitate (Table 3). Patient outcomes did not differ significantly between periods. Hospital length of stay, change in creatinine from baseline represented as percent change from baseline creatinine, and number of intraoperative and PACU RBC transfused were similar between periods (Tables 3 and 4). Similarly, postoperative mortality at 30-day and 1-year mortality was also similar (Table 4). However, surprising overall total fluid costs in the operating room and the PACU in 2013 dollars were significantly greater in the group in 2010 - Voluven® period compared to the 2009 - Hextend® period ($122.1 compared to $68.03; *P* <0.001) (Table 5). After adjusting for age, BMI, length of surgery, and type of anesthetic, Voluven® was still associated with increased fluid costs. The *R*^2^ for this multivariable model was 0.379, and the difference between periods remained significant with *P* < 0.001. Age, COPD, history of hypertension, and preoperative creatinine were also tested but dropped as non-significant effects. Results of the confirmatory analyses comparing all pre- vs. post-conversion cases were very consistent with the primary results.

**Table 3:** **Fluid volumes given**

	**2009 (** ***n*** **= 2,098)**			**2010 (** ***n*** **= 2,790)**			***P*** **value**
	**Total** ***N***	**Mean or** ***n***	**SD or percentage**	**Total** ***N***	**Mean or** ***n***	**SD or percentage**	
Hextend intraop (ml) (mean, SD)	1,906	767	301.8				
Hextend given in PACU (ml) (mean, SD)	51	578.4	215				
Hextend entire case (ml) (mean, SD)	1,941	773.1	306.9				
Voluven given intraop (ml) (mean, SD)				2,650	905.8	495.5	
Voluven given in PACU (mean, SD)				263	645.3	260.7	
Voluven entire case (ml) (mean, SD)				2,772	933.7	518.4	
Plasmanate total (mean, SD)	220	467.4	346.9	31	460.5	267.8	0.5029
Lactated Ringer’s intraop (mean, SD)	1,861	2,232.7	1,324.7	2,591	1,943.7	1,098.5	<0.0001
Lactated Ringer’s PACU (mean, SD)	87	536.2	432.1	104	471.6	388.1	0.2710
Tot lac. Ringer’s (ml) (mean, SD)	1,865	2,252.9	1,332.5	2,596	1,958.9	1,106.2	<0.0001
Normal saline intraop (mean, SD)	335	932.7	766.9	488	940	739.4	0.7984
Normal saline PACU (mean, SD)	12	175.3	158.1	45	194	205.7	0.8882
Tot N saline (ml) (Mean, SD)	344	914.5	767.4	524	892.1	744.5	0.5633
RBC’s intraop (mean, SD)	388	1,166.7	1,406.3	505	1,058.3	1,084.5	0.4291
RBC’s PACU (mean, SD)	72	597.9	313.6	82	557.6	227.9	0.8751
RBC’s total (mean, SD)	433	1,144.9	1,342.3	564	1,028.7	1,039.8	0.1790
FFP intraop (mean, SD)	123	1,069.5	1,095.3	171	947.7	938.8	0.1482
FFP PACU (mean, SD)	10	407.2	150.6	8	485.9	160.6	0.4205
Tot FFP (ml) (mean, SD)	131	1,035.3	1,071.2	177	937.5	926.6	0.2260
Cryoprecipitate intraop (mean, SD)	10	161.6	68.5	30	149.5	94.3	0.3885
Cryoprecipitate total (mean, SD)	10	161.6	68.5	31	147.9	93.1	0.3502

Intraop, intra-operative; ml, milliliters; PACU, post-anesthesia care units; Tot, Total; ml, milliliters; Lac, lactated Ringer’s; N, normal; RBC, red blood cell; FFP, fresh frozen plasma.

**Table 4:** **Health-care outcomes**

	**2009 (** ***n*** **= 2,098)**			**2010 (** ***n*** **= 2,790)**			***P*** **value**
	**Total** ***N***	**Mean or** ***n***	**SD or percentage**	**Total** ***N***	**Mean or** ***n***	**SD or percentage**	
Any PACU time (*n*,%)	1,977	1,498	75.8	2,774	2,053	74	0.1681
PACU time (hrs, incl. 0) (mean, SD)	1,977	3.2	3.3	2,774	3.2	3.3	0.0848
PACU hrs if > 0 (mean, SD)	1,498	4.3	3.2	2,053	4.3	3.2	0.2991
Any ICU stay (*n*,%)	1,971	339	17.2	2,774	629	22.7	<0.0001
ICU days	1,971			2,774			<0.0001
None (n,%)		1,632	83		2,145	77	<0.0001
1 day (*n*,%)		137	7		247	9	0.0150
2 to 3 days (n,%)		114	6		189	7	0.1530
4+ days (*n*,%)		88	4		193	7	0.0003
Total ICU days (incl. 0) (mean, SD)	1,971	0.9	4.8	2,774	1	4.2	<0.0001
ICU days if > 0 (mean, SD)	339	5.2	10.7	629	4.5	7.8	0.3676
Highest postop creatinine (mean, SD)	1,901	1.2	1	2,634	1.4	1.5	0.0884
Creatinine pre-post percentage change (mean, SD)	1,310	24.1	69	1,735	24.1	63.6	0.4533
LOS (days) (mean, SD)	1,977	7.3	9.2	2,774	7.3	9.6	0.8711
Death - 30 days (*n*,%)	1,977	32	1.6	2,774	49	1.8	0.6981
Death - 1 year (*n*,%)	1,977	164	8.3	2,774	238	8.6	0.7286

PACU, post-anesthesia care units; postop, postoperative; hrs, hours; SD, standard deviation; ICU, intensive care unit; creatinine pre-post, creatinine pre-surgery post-surgery; LOS, length of stay.

**Table 5:** **Direct fluid acquisition costs in the Hextend® and Voluven® groups**

	**2009 (** ***n*** **= 2,098)**			**2010 (** ***n*** **= 2,790)**			***P*** **value**
	**Total** ***N***	**Mean**	**SD**	**Total** ***N***	**Mean**	**SD**	
Hextend units cost	2,098	$55.13	$27.80	2,790	$0.00	$0.00	<0.0001
Voluven units cost	2,098	$0.00	$0.00	2,790	$116.01	$65.05	
Plasmanate units cost	2,098	$8.07	$29.85	2,790	$0.85	$9.21	<0.0001
LR units cost	1,977	$4.84	$2.93	2,774	$4.31	$2.52	<0.0001
NS units cost	1,977	$0.60	$1.49	2,774	$0.64	$1.53	0.196
Colloids (Hex/Vol + Plas) cost	2,098	$63.20	$33.53	2,790	$116.87	$64.80	<0.0001
All fluids units cost (observed)	1,977	$68.03	$33.22	2,774	$122.10	$65.85	<0.0001
All fluids units cost (imputed)	2,098	$68.65	$34.68	2,790	$121.82	$65.82	

Cost ($) statistics include all cases (none given = $0). ml, milliliters; LR, lactated Ringer’s; NS, normal saline; SD, standard deviation.

## Discussion

In this study, we had expected that the switch from Hextend® to Voluven® would be associated with overall decreased fluid acquisition costs due to lower utilization of albumin, an improved profile on intravascular coagulation and less need for other intravascular volume expanders as well as blood products. However, although patient demographics and outcomes were similar between trial periods, in both unadjusted and adjusted analyses, overall costs associated with the 2010 - Voluven® period were greater than the 2009 - Hextend® period. The lower cost of Plasmanate/albumin was more than offset by the considerably higher unit cost of Voluven® coupled with a higher volume of Voluven® given per case.

As many hospitals in the US are receiving bundled payments for surgical procedures, the overall hospital costs associated with surgery is becoming more important to define [15-17]. This is the first study to present the effects of a switch from Hextend® to Voluven® on fluid costs to the hospital during the perioperative period. Many surgical groups are using enhanced recovery after surgery (ERAS) protocols in colorectal and other surgeries which recommend giving a semisynthetic colloid as a plasma volume expander in the intraoperative goal-directed fluid therapy algorithm in an effort to reduce total crystalloid use [18]. The use of colloid in combination with crystalloid has been shown to decrease postoperative bowel dysfunction, including incidence of postoperative nausea and vomiting, use of rescue antiemetic, as well as pain and edema symptoms in major surgery [2]. Although our study showed an increased direct acquisition cost to the hospital in 2010 - Voluven® over 2009 - Hextend® period. A recent study at this institution suggests that goal-directed fluid therapy in which Voluven® was used as part of an ERAS algorithm resulted in a 1-L decrease in crystalloid use overall and earlier return to bowel function in patients undergoing bowel surgery as well as a 2-day reduction in hospital length of stay [19]. Whether the use of a plasma expander or other parts of the ERAS bundle contributed to this decreased length of stay needs further study. We did not target a specific population in our study, and future studies should specifically focus on this area of investigation and question which part of the ERAS bundle leads to the most beneficial patient outcomes.

Several limitations to the current study need consideration. This is a single center study, and hence, the results may not be generalizable to other institutions or other countries given differences in fluid acquisition costs and availability depending on regulatory agencies. Also, given the retrospective nature of this study, unaccounted confounders may exist which may have skewed our results. However, because baseline demographics are similar as the types of surgeries, this is less likely to be a major issue.

## Conclusions

In summary, after converting from Hextend® to Voluven® in a large tertiary medical institution, no differences were seen in short- and long-term mortality, renal outcome, blood and blood product utilization, ICU, or hospital length of stay. Although the number of cases receiving albumin decreased, total intraoperative and PACU intravascular volume fluid acquisition costs were greater.

## Abbreviations

CI: confidence interval
ERAS: enhanced recovery after surgery
ICU: intensive care unit
kD: kilodalton
ml: milliliter
MW: molecular weight
PACU: post-anesthesia care unit
RBC: red blood cells
SD: standard deviation
